# Effects of whole body vibration exercise combined with weighted vest in older adults: a randomized controlled trial

**DOI:** 10.1186/s12877-022-03593-4

**Published:** 2022-11-28

**Authors:** Lertwanlop Srisaphonphusitti, Nuttaset Manimmanakorn, Apiwan Manimmanakorn, Michael John Hamlin

**Affiliations:** 1 Exercise and Sports Science Program, Graduate School, Khon Kaen University, Khon Kaen, Thailand; 2 Research Center in Back, Neck, Other Joint Pain and Human Performance (BNOJPH), Khon Kaen University, Khon Kaen, Thailand; 3 Department of Rehabilitation Medicine, Faculty of Medicine, Khon Kaen University, Khon Kaen, Thailand; 4 Department of Physiology, Faculty of Medicine, Khon Kaen University, Khon Kaen, Thailand; 5 Department of Tourism, Sport and Society, Lincoln University, Christchurch, New Zealand

**Keywords:** Balance, Functional mobility, Muscle strength, Elderly people, Vest, Vibration

## Abstract

**Background:**

To evaluate the training effects of whole body vibration (WBV) combined with weighted vest (WV) in older adults.

**Methods:**

This randomized controlled trial study was conducted in healthy older adults living in the community. Fifty-one participants were randomly allocated into 3 groups: group 1 (*n* = 17), WBV alone, training on WBV at a frequency 30 Hz, amplitude 2 mm, 10 sets of 1 min squats, with 60 s rest, group 2 (*n* = 15), WV alone, squat exercise, 10 sets of 1 min, with 60 s rest, while WV loaded with 10% body weight and group 3 (*n* = 19), WBV + WV, combining WBV exercise with the addition of a WV. All groups completed training 3 times per week for 8 weeks. The outcomes were total muscle mass, muscle thickness, maximal isometric strength, single-leg-stance and timed-up-and-go evaluated at baseline and after training.

**Results:**

As a result of training all groups improved their isometric muscle strength with little difference between groups. The single-leg-stance significantly improved only in WBV + WV group 25.1 ± 10.8 s (mean ± 95% CI, *p* < 0.01). The timed-up-and-go improved in all groups, but the improvement was significantly greater in the WBV + WV group (17.5 ± 6.9%) compared to the WV (8.5 ± 3.2%) and WBV groups (9.2 ± 5.4%, *p* = 0.043, 0.023 respectively). Rectus femoris muscle thickness and total muscle mass were significantly increased in all groups equally with little difference between groups.

**Conclusion:**

The combined WBV + WV had a greater effect on the single-leg-stance and the timed-up-and-go compared to WV or WBV alone.

**Trial registration:**

TCTR20190306001. Thai Clinical Trials Registry (www.thaiclinicaltrials.org). Date of registration: 6 March 2019.

**Supplementary Information:**

The online version contains supplementary material available at 10.1186/s12877-022-03593-4.

## Background

Whole body vibration (WBV) exercise was recently suggested as a safe and effective substitute for conventional exercise in older adults [1]. WBV exercise provides vibration transmitted from a platform underneath the participants feet to stimulate muscle contraction via tonic vibration reflexes [2]. In older adults, WBV exercise was found to improve bone mass, muscle strength, balance, reduce risk of falls and increase quality of life [3–7]. In a meta-analysis on the effect of WBV exercise in older adults, Lau et al. (2011) found significant positive effects on knee extension strength, jumping height and performance in the sit-to-stand test [6]. WBV exercise also produces a small to moderate positive effect on static and dynamic balance in older adults [8, 9].

Weighted vests (WV) are upper body clothing garments that can provide more resistance loading to exercise by providing additional weight in the vest. Like WBV exercise, WV have been used during exercise training with older adults with some success. Shaw et al. (1998) found that resistance exercise for 9 months with a WV improved muscle strength and balance in older adults compared to a conventional activity control group [10]. Klentrou et al. (2007) had postmenopausal women complete multimodal exercise for 65 min per day, 3 days per week over 12 weeks, wearing a WV (progressively increased up to 15% of participants body weight), and found a significant reduction in serum osteocalcin levels (a bone resorption marker) and improved ankle plantar-flexion strength [11]. In addition, Mierzwicki (2019) revealed 12 weeks of lower extremity exercises with WV loading equivalent to 10% of participants bodyweight improved hip muscle strength, 30-sec chair rise,2-min step and 6-min walk test performance in older adults [12].

The combination of WBV exercise with additional load aimed at increasing exercise workload has been found to facilitate muscle activity in young adults [13, 14], and increase VO_2_ and energy expenditure in young adults and older adults [15, 16]. Adding load via barbell exercises or Olympic bar lifts to WBV exercise results in improved muscular power, speed and agility in young athletes [17] but such loads would be considered unsafe for weaker, older individuals. The combination of WBV exercise with additional load in a WV may be safe and suitable for older adults to increase muscle strength and body balance more than WBV or WV alone. The acute effects of combining these two exercise modes in older adults have been recently published [18], however, training effects of these combined exercises in older adults have not been reported.

Therefore, the aim of this study was to examine the training effects of adding extra load (by the use of WV in an attempt to increase total workload) to WBV exercise (WBV + WV) on muscle strength and body balance in older adults.

## Methods

### Participants

The study was an assessor-blinded randomized controlled trial with parallel group design. This study was conducted at Department of Rehabilitation Medicine, Srinagarind Hospital, Faculty of Medicine, Khon Kaen University, Thailand. The period of the study was from March 2019 to June 2019. The included participants were healthy older adults, aged 60-80 years and lived in the community. The participants were examined by a physician and completed a medical health questionnaire before enrolling in this study. The participants were excluded if they had any of the following conditions including musculoskeletal problems such as severe back pain, hip and ankle pain, lumbar spondylosis, spinal stenosis, osteoporosis, uncontrolled medical conditions such as diabetes, hypertension or heart diseases, and uncontrolled psychological disorders.

**Fig. 1:**
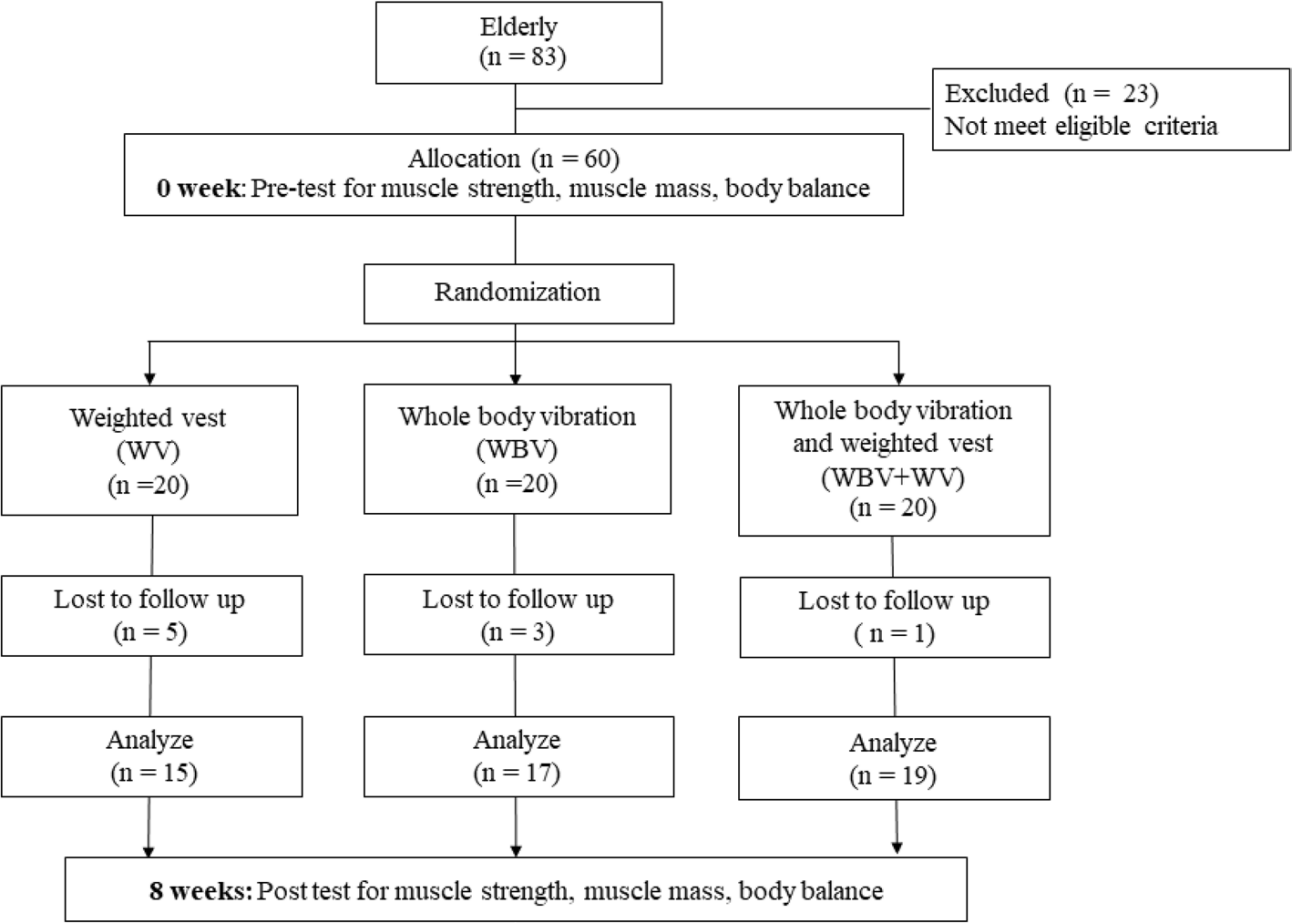
Consort diagram of the study

After giving informed consent, sixty participants who were enrolled in this study by authors LS and NM were randomized to alternative exercise programs on a 1:1:1 ratio by using block of 6 design. The random allocation sequence was generated by computer program and the random sequence was concealed by opaque sealed envelopes. The randomization and allocation of participants to the various groups was conducted by a research assistant who was not involved in the current study, was blinded to the group details, and was not influenced by any of the researcher’s conducting this study. The participants were allocated into 3 groups (*n* = 20/group): group 1, WBV alone; group 2, WV with additional load at 10% of body weight; and group 3, WBV combined with WV with additional load at 10% of body weight. There were 3 participants in WBV group, 5 participants in WV group and 1 participant in WBV + WV group who dropped out because of time constraints (Fig. 1). Therefore, fifty-one healthy older adults, 11 males and 40 females, were included in this study. The average age was 65**.6** ± 3.8 years (mean ± SD) with an average BMI of 25.9 ± 3.9 kg.m^− 2^.

This study was carried out in accordance with the Declaration of Helsinki and was approved by the local ethical board (Khon Kaen University Human Ethical Committee No. HE611192).

### Interventions

In the WBV group, the participants were trained on a synchronized vibration platform (Power Plate® Pro5, Performance Health Systems UK Ltd., London, UK) at a frequency 30 Hz, amplitude 2 mm, 10 sets of 1 min, interspersed with 60 s upright standing rest, 3 times a week for 8 weeks. The participants squatted on the platform without shoes holding knee flexion of approximately 20^o^. In WBV + WV group, the participants completed the same training as the WBV groups except the participants also wore a WV (Domyos, Decathlon, Villeneuve d’Ascq France) on the upper part of body. Small bags of sand were inserted into the pockets around the vest for an evenly distributed additional weight. The weight in the vests was progressively increased as follows: 1st week no load (just the weight of the vest which was approximately 0.5 kg), 2nd week an additional 5% body weight and 3rd week an additional 10% bodyweight through to 8 weeks. In WV only group, the participants completed the same squat exercises as the WBV and WBV + WV groups with a knee flexion of 20^o^ on a solid and stable surface (10 sets of 1 min, interspersed with 60 s rest periods), while wearing vests loaded with the extra weight. The extra weight in WV only group was increased progressively in a similar fashion to the WBV + WV group. Compliance to training and adverse effects were recorded. The participants were reimbursed transportation costs each time they turned up to the training exercise. During the experiment, the participants were asked to maintain their normal lifestyle and physical activity levels.

### Outcome measurements

The primary outcome of this study was maximal isometric muscle strength, and the secondary outcomes were total body mass, muscle thickness, body balance (single-leg-stance test) and functional mobility (timed-up-and-go test). Outcome measurements were evaluated at baseline and after 8-weeks training (1 day after the last training session). The researchers who assessed the outcomes were blinded to group assignment. The performance measures were performed after a warm-up exercise of light isotonic exercises with a full range of motion of both the upper and lower extremities for 3 min. The participants performed a familiarization test 1 week prior to the experiment. The performance testing started with muscle strength followed by the single-leg-stance test and then the timed-up-and-go test with 5 min recovery between each test. The best of three repeated trials with a 5 min rest period between trials was analyzed.

Isometric muscle strength of the trunk and leg muscles was evaluated by a back and leg dynamometer (TKK 5002, Takei Scientific Instruments, Niigata, Japan). The dynamometer-measured isometric muscle strength is a highly reliable measurement and has a strong correlation to knee muscle strength [19]. The chain length on the dynamometer was adjusted so that when the participants stood on the base of the unit, they produced a knee flexion of 110^o^, hips in slight flexion, back slightly forward and head in the upright position. The participants were asked to lift the handle (positioned at the line of the knee joint) in a vertical direction by using their leg muscles as much as possible with no back bending. The participants were asked to gradually increase the isometric force to maximum level over a 3 s period.

Static body balance in older adults was assessed by a single-leg-stance test that has shown excellent test-retest reliability and discriminant validity [20]. Before testing, the participants were instructed to stand on their preferred leg for as long as possible without any hand-held support. Standing time (in seconds) was recorded from when 1 foot was lifted off the floor and ended when the same foot touched the ground or the other leg.

Functional mobility was tested with the timed-up-and-go test which has excellent reliability and validity in older adults [20]. The participants after sitting comfortably in a chair were instructed to get up from the chair, walk with a comfortable speed to a point on the floor 3 m away. The participants then turned around at the 3-m point, and walked back to the chair, and sat down to the starting position. Recording time was started when the participant started to rise out of the chair and stopped when finally back in the original sitting position.

The composition of body was assessed using bioelectrical impedance analysis (Tanita Pro BC-418, Tanita Corporation, Tokyo, Japan). The participants stood barefoot on the 2 foot-pad electrodes mounted on a platform. The measurement was performed at the same time in the morning, around 2 hours after eating breakfast, and within 30 min of emptying the participants bladder. Participants were asked to refrain from any high-intensity exercise for at least 24 hours prior to testing. The bioelectrical impedance analyzer estimated fat mass, fat free mass, body fat percentage and muscle mass at pre and post training.

The thickness of rectus femoris and gastrocnemius muscles were evaluated by using ultrasonography with a 9 MHz linear transducer (LOGIQ e; General Electric, Co., Ltd., USA). The measurement was performed twice using a blinded assessor, (a rehabilitation medicine specialist). The average of two assessments (separated by a 5 min period) was recorded and analyzed. The thickness of the quadriceps muscle was measured on the anterior aspect of thigh at the midpoint between the anterior superior iliac spine and the medial femoral condyle, while the participants rested in supine position on the bed with hips and knees extended. The thickness of gastrocnemius muscle was measured at midpoint between popliteal crease and the calcaneal tuberosity, measured in the prone position with knees extended and exactly at the midpoint of the girth of the muscle. Muscle thickness measurements were taken on the day following strength, balance and functional mobility testing. Participants were tested in the morning approximately 2 hours after eating breakfast and after 30 min of emptying their bladders. Participants were asked not to consume anything between breakfast and the muscle thickness test.

Heart rate was monitored by PolarTeam Pro Sensor (Polar Electro, Kempele, Finland). The sensor attached with chest strap was placed on the mid anterior chest wall at the xiphoid process level. Blood pressure was measured by automatic blood pressure monitor (Omron HEM-7121, Omron Healthcare Co., Ltd., Kyoto, Japan). The heart rate and blood pressure were recorded after 15 min rest in the morning at the same time of day, before and after 8 weeks post-training.

### Statistical analysis

Statistical analysis was performed using IBM SPSS statistical analysis package (version 26, SPSS, Inc., Chicago, IL, USA). The descriptive data is presented as mean, standard deviation and changes mean and 95% confidence intervals. A Kolmogorov-Smirnov test was performed to check for the normality of the data distribution. Normally distributed data including heart rate, blood pressure, BMI and muscle thickness was analyzed for change within groups by a paired t-test and the difference between the groups was analyzed by a one-way ANOVA test with Post Hoc analysis. Non-parametric data such as fat, muscle mass, muscle strength, single leg stance and functional mobility was analyzed for changes within groups by a Wilcoxon Signed-Rank test, and the differences between the three groups was analyzed by a Kruskal-Wallis test. When the *p* value was less than 0.5, the results were considered statistically significant.

The sample size was estimated based on a three-group comparison with a power of 80% (beta error 0.2) and a statistical significance *p* < 0.05 (alpha error 0.05). The variance of the main outcome (muscle strength) from a previous study [14] was 1 kg and the estimated desired difference between experimental and control group was 1 kg. Therefore, 16 participants per group were required, after correcting for a dropout rate of approximately 20%, the total participants for 3 groups were 60.

## Results 

The characteristics of the participants in each group are presented in Table 1. There was no significant difference in the characteristics between the participants in the three groups at baseline (WV, WBV, and WBV + WV). On average, participants made 90% of the exercise training times. No adverse effects of training such as back or leg pain were observed throughout the study in any group.

**Table 1:** Characteristics and physiological variables of participants in the three groups

Characteristics	WV (*n* = 15)	WBV (*n* = 17)	WBV + WV (*n* = 19)	*p*-value
Age (years)	67.0 ± 4.8	65.3 ± 3.6	64.5 ± 3.1	0.075
Gender (male/female)	3/12	2/15	6/13	0.396
Height (cm)	152.5 ± 7.5	154.3 ± 6.5	154.4 ± 8.0	0.738
Weight (kg)	59.0 ± 10.4	61.9 ± 10.4	63.6 ± 9.6	0.448
Body mass index (kg.m^− 2^)	25.2 ± 3.1	26.0 ± 4.4	26.7 ± 4.6	0.580
Resting heart rate (bpm)	78.1 ± 9.1	76.8 ± 8.0	7 7.6 ± 8.1	0.243
Systolic blood pressure (mmHg)	120.5 ± 14.4	121.8 ± 13.8	123.5 ± 9.9	0.787
Diastolic blood pressure (mmHg)	74.1 ± 9.1	70.5 ± 9.0	77.0 ± 6.5	0.072

Data are mean ± SD

*WV* weighted vest (10% of body weight), *WBV* whole body vibration, *WBV + WV* whole body vibration combined with weighted vest (10% of body weight)

Compared to baseline, 8 weeks of training had little effect on resting heart rate, systolic or diastolic blood pressure or fat mass in any group, but increased muscle mass equally (ranged from 0.6-1.0 kg) in all groups as measured by the bio-electrical impedance device (Table 2).

**Table 2:** Heart rate, blood pressure and body composition before and after 8 weeks of training in the three groups

Outcomes	WV (*n* = 15)			WBV (*n* = 17)			WBV + WV (*n* = 19)			
	Pre-test	Post-test	Mean difference ± 95%CI	Pre-test	Post-test	Mean difference ± 95%CI	Pre-test	Post-test	Mean difference ± 95%CI	*p*-value between 3 groups
Heart rate (bpm)	78.1 ± 9.1	76.7 ± 8.2	-1.4 ± 2.6	76.8 ± 8.0	73.3 ± 8.7	−3.5 ± 4.1	77.6 ± 8.1	73.3 ± 8.7	−4.3 ± 4.3	0.268
Systolic blood pressure (mm Hg)	120.5 ± 14.3	115.5 ± 12.4	−5.1 ± 6.7	121.7 ± 13.7	120.6 ± 12.7	−1.1 ± 6.7	123.5 ± 9.9	121.2 ± 10.6	− 2.4 ± 4.7	0.625
Diastolic blood pressure (mm Hg)	74.1 ± 9.1	70.7 ± 9.3	− 3.4 ± 4.5	72.1 ± 6.9	71.1 ± 7.8	−1.0 ± 5.0	77.0 ± 6.4	75.5 ± 9.3	−1.5 ± 5.4	0.772
Body mass index (kg.m^− 2^)	25.2 ± 3.1	25.2 ± 3.2	− 0.0 ± 0.3	26.0 ± 4.3	25.9 ± 4.4	− 0.1 ± 0.3	26.6 ± 4.0	26.5 ± 4.0	− 0.1 ± 0.2	0.185
Fat (%)	33.6 ± 6.5	33.5 ± 7.1	− 0.1 ± 1.6	34.7 ± 1.0	34.1 ± 10.3	−0.6 ± 0.7	34.3 ± 10.6	33.6 ± 10.6	−0.7 ± 0.8	0.659
Fat mass (kg)	20.0 ± 6.0	19.8 ± 6.5	−0.2 ± 0.7	22.1 ± 9.1	21.6 ± 9.2	−0.4 ± 0.6	22.2 ± 9.1	21.5 ± 8.7	−0.7 ± 0.7	0.600
Muscle mass (kg)	36.9 ± 7.0	37.5 ± 7.0	0.6 ± 0.4*	36.9 ± 5.8	37.7 ± 5.5	0.8 ± 1.3*	38.5 ± 6.5	39.5 ± 6.7	1.0 ± 1.1*	0.227

Data are mean ± SD for pre-test and post-test and mean ± 95% confidence interval for post-pre test change

*WV* weighted vest (10% of body weight), *WBV* whole body vibration, *WBV + WV* whole body vibration combined with weighted vest (10% of body weight)

*Significant difference compared within group (pre vs post), *p* < 0.05

As a result of training, all 3 groups improved their isometric lower body strength (improvement ranged from 17.2 to 19.4 kg), with little difference between groups (Table 3). Likewise, balance (single-leg-stance) improved in all groups (ranged from 10.3 to 25.1 s). However, the increase in balance was only statistically significant in the WBV + WV group (25.1 ± 10.8 s) Table 3. Functional mobility measured by the timed-up-and-go test, also improved in all groups as a result of training (ranged from 0.7 − 1.4 s), and the improvement in this measure was significantly greater in the WBV + WV group compared to the WV and WBV groups (Table 3).

**Table 3:** Performance variables before and after 8 weeks of training in the three groups

Outcomes	WV (*n* = 15)			WBV (*n* = 17)			WBV + WV (*n* = 19)			
	Pre-test	Post-test	Mean difference ± 95%CI	Pre-test	Post-test	Mean difference ± 95%CI	Pre-test	Post-test	Mean difference ± 95%CI	*p* -value between 3 groups
Muscle strength (kg)	73.7 ± 31.0	90.9 ± 46.5	17.2 ± 12.0^*^	78.3 ± 25.4	97.4 ± 27.4	19.1 ± 7.0^*^	82.9 ± 30.0	102.3 ± 34.6	19.4 ± 5.8^*^	0.913
Single leg stance (s)	90.3 ± 87.0	115.0 ± 105.9	24.7 ± 50.6	92.6 ± 84.2	102.9 ± 61.3	10.3 ± 31.4	71.6 ± 54.6	96.7 ± 62.5	25.1 ± 10.8^*^	0.731
Timed up and go (s)	7.9 ± 1.6	7.1 ± 1.2	− 0.7 ± 0.4^*,a^	7.2 ± 1.0	6.6 ± 0.9	−0.6 ± 0.2^*,b^	8.2 ± 1.8	6.7 ± 0.9	− 1.4 ± 0.6^*,a, b^	0.043*

Data are mean ± SD for pre-test and post-test and mean ± 95% confidence interval for post-pre test change

*WV* weighted vest (10% of body weight), *WBV* whole body vibration, *WBV + WV* whole body vibration combined with weighted vest (10% of body weight)

*Significant difference compared within group (pre vs post), *p* < 0.05

^a^Significant difference compared between WV and WBV + WV

^b^Significant difference compared between WBV and WBV + WV, *p* < 0.05

Muscle thickness as measured by the ultrasound technique was significantly increased as a result of training in all groups equally (ranged from 2.0 to 4.0 mm in the right rectus femoris and 2.3 to 2.7 mm in the left rectus femoris, Table 4). Conversely, 8 weeks of training had little effect on the gastrocnemius muscle thickness in any group (Table 4).

**Table 4:** Muscle thickness before and after 8 weeks of training in the three groups

Muscle thickness (mm)	WV (*n* = 15)			WBV (*n* = 17)			WBV + WV (*n* = 19)			
	Pre-test	Post-test	Mean difference ± 95%CI	Pre-test	Post-test	Mean difference ± 95%CI	Pre-test	Post-test	Mean difference ± 95%CI	p- value between 3 groups
Rectus femoris muscle (R)	30.5 ± 4.9	32.5 ± 5.9	2.0 ± 1.7*	27.0 ± 3.4	29.8 ± 4.1	2.8 ± 1.8*	26.7 ± 4.0	30.7 ± 4.1	4.0 ± 2.4*	0.333
Rectus femoris muscle (L)	28.7 ± 4.8	31.0 ± 4.3	2.3 ± 1.6*	26.8 ± 3.5	29.4 ± 4.7	2.6 ± 2.4*	27.3 ± 3.8	30.0 ± 5.3	2.7 ± 2.4*	0.984
Gastrocnemius (R)	26.1 ± 5.9	25.2 ± 3.0	−0.9 ± 3.3	26.8 ± 4.3	26.3 ± 4.6	−0.5 ± 1.6	25.5 ± 4.8	24.4 ± 2.7	− 1.1 ± 1.0	0.906
Gastrocnemius (L)	26.0 ± 5.1	25.3 ± 2.9	− 0.7 ± 2.9	25.6 ± 3.3	26.6 ± 3.5	1.0 ± 1.9	25.5 ± 5.2	24.7 ± 3.0	− 0.8 ± 0.7	0.435

Data are mean ± SD for pre-test and post-test and mean ± 95% confidence interval for post-pre test change

*WV* weighted vest (10% of body weight), *WBV* whole body vibration, *WBV + WV* whole body vibration combined with weighted vest (10% of body weight)

*Significant difference compared within group (pre vs post), *p* < 0.05

## Discussion

It is well understood that many countries will have a substantial increase in their older age group population over the next 10-20 years. This “greying” of society requires special attention, particularly to interventions that aim to reduce the chronic diseases in this population. Subsequently, new and effective ways to improve health and reduce disease burden in this population are required. This study has revealed that a unique physical activity intervention was effective at improving health (increased balance, improved strength and better functional mobility), in an older age group which may result in improved wellbeing.

This study revealed WBV exercise at low frequency (30 Hz) and low amplitude (2 mm) for 8 weeks increased overall back and lower extremity muscle strength and functional mobility (timed-up-and-go test), but had little effect on static balance (single-leg-stance test). Previous meta-analytical studies revealed the beneficial effect of WBV. Lau et al. (2011) conducted a meta-analysis on the effect of WBV (frequency 10-54 Hz, amplitude 0.05-8 mm, 1-7 session per week, duration 6 weeks to 18 months) in older adults and found significant positive effects on knee extension isometric strength, jumping height and performance in the sit-to-stand test [6]. The possible mechanism causing WBV to increase muscle strength can be explained by the vibratory tonic reflex. The vibration through the body stimulates type Ia afferent fibers and consequently enhances the α-motor neurons to activate muscle activity [2]. WBV also changes the hormonal levels such as cortisol, testosterone and growth hormone [21, 22]. Two previous meta-analysis studies were conducted in older adults and reported significant benefits of WBV on static and dynamic balance [8, 9]. It seems that vibration training can improve muscular strength and stimulate joint proprioception sensation [23] which thereby influences functional mobility, static balance and increases performance in tests like the timed-up-and-go.

Our study found that completing a squatting exercise 3 times per week for 8 weeks while wearing a WV improved back and lower extremity strength, quadriceps muscle thickness and functional mobility. The results of this study are similar to previous studies that have suggested beneficial effects of training while wearing WV in older adults. Shaw et al. **(**1998**)** found that 60 min of weight-bearing exercise **(**35 min lower extremities resistance exercise**)** with progressive additional WV **(**16-20% of body weight**)** for 9 months in older women improved muscle strength **(**knee extension, hip abduction and ankle plantar flexion**)** and dynamic balance (dynamic posturography) compared to customary activity [10]. Klentrou et al. **(**2007**)** also found that 65 min of multimodal exercise while wearing a WV **(**maximum of 15**%** body weight for 12 weeks**)** in postmenopausal women decreased a bone resorptive marker and increased ankle plantar-flexion strength compared to normal controls [11]. Adding resistance to exercise (in our case by adding sand in the vest), results in an increased stress on the working muscles used during movement. As a consequence, a greater amount of force is required to be generated by the working muscles either through an increase in the number of active motor units and/or an increase in their firing frequency [24]. Over time through adaptation to this increased stress on the skeletal muscles, hypertrophy occurs, and muscle cross-sectional area increases [25]. We found an increase in strength in the WV group of approximately 23% which coincided with an increase in muscle thickness (a surrogate for cross-sectional area) of approximately 7-8% indicating that at least some of the improvement in strength was associated with an increase in quadriceps muscle thickness.

WBV exercise with WV increased overall back and lower extremity strength, rectus femoris muscle thickness, single-leg-stance balance time and speed in the timed-up-and-go test compared to baseline and significantly improved performance in the single-leg-stance balance test and the timed-up-and-go test compared to WV and WBV training alone. In a previous study the combination of vibration and extra-load training on athletes also found such training improved muscular strength compared to an unloaded vibration or loaded training group [26]. But this is the first study to show that vibration with extra-load training can not only improve strength, but also balance in a group of older adults.

The addition of weighted resistance combined with WBV enhanced the muscle hypertrophy of the rectus femoris muscles (as indicated by the muscle thickness measures) resulting in slightly more muscle strength but significantly greater improvement in functional mobility (timed-up-and-go test) than WV or WBV alone. The additional load during WBV exercise stimulates muscle activity [13, 14] and energy expenditure [15, 16] to a higher level than training WBV alone. We postulate that the WV training was a low-intensity type of training that probably required only Type I muscle fibers to accommodate the force required. However, it is known that WBV exercise stimulates Type II muscle fibers [27], therefore adding resistance to WBV exercise (as in the combined group in this study) may increase the stress on the muscles and shift the recruitment towards Type II muscle fibers, thereby resulting in hypertrophy and muscle strength gains. However, this is speculative and would require further research using EMG techniques before we could confirm such a mechanism.

Balance and postural control are very important for older adults to prevent falling. The single-leg-stance test is used for measuring static balance and postural control that is correlated to muscle strength and joint proprioception [28, 29]. The single-leg-stance test is an important predictor of fall risk or fall injury risk in older adults [30]. This study revealed the combined WBV and WV exercise group had the greatest improvement in the single-leg-stance test that may provide the greatest enhancement of muscle strength and joint proprioception. This study found no significant muscle strength improvement between the three groups, while joint proprioception may be more enhanced by combination training. However, joint proprioception was not evaluated in this study. The combination training improved static balance to a greater degree in our participants, which may result in less risk of falling in this group.

This study found the greatest improvement in the timed-up-and-go test after the combined WBV and WV exercise. The timed-up-and-go test was used to indicate functional mobility in our study which is related to a number of factors such as muscle strength, muscle power, balance, mobility and aerobic capacity [31]. Previous research has shown that the timed-up-and-go test is an effective screening tool for indicating risk of falls in frail older adults [32]. While this study was conducted on healthy older adults, if such training also improves the timed-up-and-go performance in older frail adults it may protect these older participants from future falls.

This study revealed fat mass, %fat and BMI did not significantly change from baseline or between groups. We speculate the training duration was probably too short to see any substantial changes in fat mass. Additionally, this study found little change in blood pressure or resting heart rate in the three groups. Again, we speculate that the squatting exercise in WV, WBV or WBV + WV was not intensive enough to produce significant cardiovascular change [33]. In addition, our study was conducted on participants with normal blood pressure, and previous studies have suggested such exercise may be more beneficial for prehypertension and hypertension patients [34, 35].

The strength of study is the research design which was a randomized controlled trial which used blinded assessors to conduct the outcome measurements. The outcomes included both laboratory and functional outcomes that were relevant to the health problems of older adults. One limitation of this study is generalizability since we used active healthy older adults. Future research should incorporate similar training interventions on older adults with health problems. A further limitation was the fact that we used low frequency with low amplitude vibration training which may not be the most suitable for this age group. Therefore, future research should investigate which vibration parameters are the most effective for training in older adults. The study was also limited in terms of measurement outcomes which included muscle strength and balance. Other outcomes such as hormonal changes or bone density should be also measured in the future studies.

## Conclusion

All three groups improved muscle strength, muscle thickness, total muscle mass, single-leg-stance ability, and the timed-up-and-go time as a result of training. However, after 8 weeks we found that WV + WBV had a greater beneficial effect on the single-leg-stance and timed-up-and-go tests. Combined WBV with a WV exercise may therefore be recommended as an alternative exercise training protocol for improving muscle strength, performance and body balance in older adults.

## Supplementary Information

**Additional file 1.**

## Data Availability

The datasets used and/or analyzed during the current study are available. from the corresponding author on reasonable request.

## Abbreviations

WBV: Whole body vibration
WV: Weighted vest
BMI: Body mass index
CI: Confidence interval
EMG: Electromyography
